# Genetic variation of *NEDD4L *is associated with essential hypertension in female Kazakh general population: a case-control study

**DOI:** 10.1186/1471-2350-10-130

**Published:** 2009-12-09

**Authors:** Nanfang Li, Hongmei Wang, Jin Yang, Ling Zhou, Jing Hong, Yanying Guo, Wenli Luo, Jianhang Chang

**Affiliations:** 1 The Center of Hypertension of the People's Hospital of Xinjiang Uygur Autonomous Region, The Center of Diagnosis, Treatment and Research of Hypertension in Xinjiang, No 91, Tianchi Road, Urumqi, Xinjiang, China

## Abstract

**Background:**

Hypertension affects > 18.8% of adults in China. Indeed, hypertension is the most prevalent risk factor for cardiovascular morbidity and mortality worldwide. Genetic variation is thought to contribute to the etiology of hypertension. *NEDD4L *is a candidate gene for hypertension, both functionally and genetically. The purpose of the current study was to investigate the relationship between the variation in *NEDD4L *and essential hypertension in Kazakh, which is a relatively isolated population with a pure genetic background and is an ideal population to study genetic mechanisms of hypertension.

**Methods:**

We screened the promoter and exons of *NEDD4L *in 94 Kazakh hypertensive individuals to identify representative variations. Then, by genotyping the representative variations in the Kazakh general population, a case-control study was conducted.

**Results:**

By systemically screening variations of *NEDD4L*, we did not identify any functional mutations in *NEDD4L*. A new common variation (296921-296923delTTG), which is not found in the NCBI database, was identified. Three representative variations (296921-296923delTTG, rs2288774, and rs2288775) were successfully genotyped in the Kazakh general population. The distribution of the dominant model (AA *vs. *AG+GG) of rs2288775, the additive model, and the recessive model (II+ID *vs. *DD) of 296921-296923delTTG differed significantly between the cases and controls in females (*P *= 0.040, *P *= 0.024, and *P *= 0.007, respectively). After adjusting for confounding factors, logistic regression analysis showed that rs2288775 (in the dominant model) and 296921-296923delTTG (in the recessive model) were significantly associated with hypertension (rs2288775: OR = 1.479, 95% CI = 1.011-2.064, *p *= 0.044; and 296921-296923delTTG: OR = 1.908, 95% CI = 1.020-3.568, *p *= 0.043) in females. The frequency of the D-C-G haplotype was significantly higher for cases than for controls in females (*P *= 0.020). There was a significant interaction between the *NEDD4L *genotype and gender (*P *for interaction: 0.045 for rs2288775 and 0.064 for 296921-296923delTTG), but there was no significant interaction between the *NEDD4L *genotype and smoking (*P *for interaction: 0.616 for rs2288775 and 0.447 for 296921-296923delTTG). For females and total participants, the urinary Na excretion rate was significantly lower in the DD than the I/I+I/D individuals (*P *= 0.032 and *P *= 0.027 respectively).

**Conclusion:**

The genetic variations of *NEDD4L *may be associated with essential hypertension in females in the Kazakh general population.

## Background

Hypertension affects > 18.8% of the adult population in China. Indeed, hypertension is the most prevalent risk factor for cardiovascular morbidity and mortality worldwide [1]. However, the etiology of > 90% of patients with hypertension is unknown. Studies have revealed that essential hypertension is a complex phenotype in which genetic factors explain 30%-60% of all variations in the population [2]. Thus, it is important for the prevention and treatment of essential hypertension to identify the susceptible genes in different populations.

A region on chromosome 18q21-22 has been identified in a number of genome-wide scans on hypertension, therefore making the locus a suitable candidate for harboring gene/genes involved in blood pressure (BP) regulation [3,4]. The 18q21-22 locus harbors an important BP-regulating gene (the neural precursor cell expressed developmentally down-regulated 4-like [NEDD4L] gene). NEDD4L regulates BP through its ubiquitinating effects on the epithelial sodium channel (ENaC), a critical component of the pathway maintaining salt and water balance [5] at the luminal cell membrane in the renal collecting ducts [6-8], resulting in reduction of the number of ENaC present on the plasma membrane. Peijun *et al*. demonstrated that *NEDD4L *knockout mice have high BP and an impaired ability to down-regulate ENaC activity [9]. Thus, *NEDD4L *is a candidate gene for essential hypertension. Recently, a common polymorphism located at intron 2 (rs4149601) of the *NEDD4L *gene was shown to be associated with hypertension both in African Americans and Caucasians, and a "flip-flop" association with hypertension was found in two Caucasian samples [10] for a common polymorphism located at intron 12 (rs3865418). However, there are no reports regarding the relationship between genetic variations in the human *NEDD4L *gene and essential hypertension in Kazakh, which is an ideal population to study genetic mechanisms of hypertension.

Kazakh, a nomad population which dwells north of Xinjiang in northwest China and in which 99% are herdsman, is characterized by a higher prevalence of hypertension and higher BP levels compared to other ethnic populations residing in the same area [11]. Moreover, the hypertension in this population is salt-sensitive and BP decreases significantly after limiting salt intake [11]. A previous study has confirmed that compared with other populations, including Mongolian, Hui, Tibetan, Uygur, Miao, Yi, Zhuang, Buyi, Man, Yao, and Tujia, the Kazakh population has a higher dairy intake, with an average daily consumption > 21 g [12]. Furthermore, very few Kazakh marry people of other ethnicities because the unique customs, cultural background, and food habits are different from other ethnicities. Therefore, Kazakh is a relatively isolated population with a pure genetic background and is an ideal population to study genetic mechanisms of complex diseases, such as hypertension.

Essential hypertension, as a complex trait, has been suggested to be caused by common sequence variants that may have a small-to-moderate phenotypic effect [13-15]. On the other hand, a study has shown that most Mendelian disorders are caused by a set of different rare mutations that reside in coding regions, which tend to have strong phenotypic effects. Recently, several studies have shown that rare genetic variations in *ABCA1*, *APOA1*, and *LCAT *collectively contribute to variation in plasma levels of high-density lipoprotein-cholesterol (HDL-C) in the general population [16,17]. We therefore hypothesized that not only common, but also rare genetic variations in *NEDD4L*, could contribute to hypertension.

In this study, we sequenced the promoter and all exons of *NEDD4L *in 94 Xinjiang Kazakh hypertensive individuals to identify common single nucleotide polymorphisms (SNPs) and rare variations, and then genotyped the representative variations in the general population to systemically study whether genetic variations in *NEDD4L *are implicated in essential hypertension in the Xinjiang Kazakh general population.

## Methods

### Study population

One thousand Kazakh subjects with no mixed marriages within the past three generations were randomly recruited for this study by multistage cluster sampling from the Fukang area in the Xinjiang Uygur Autonomous Region. Written consent was obtained from all subjects before any data collection and measurements. A total of 956 individuals completed the survey during the 1-month period from January to February 2008, with an overall response rate of 95.6%. Subjects with secondary hypertension, excessive drinking, cancer, age < 30 years or > 60 years, and use of contraceptives were excluded from this study, leaving a final study population of 883 subjects (375 males and 508 females) for the current set of analyses. Then, a case-control study was conducted in 383 hypertensive and 500 normotensive subjects. Secondary hypertension was excluded by history, examination, and laboratory evaluation. This study was approved by the Ethics Committee of the People's Hospital of Xinjiang.

### Diagnostic criteria and measurements

In light of a common protocol recommended by the American Heart Association Anthropometric measurements, the BP measurement was performed by trained and certified observers 3 times per subject with at least 10 min of rest in a sitting position and the mean value was considered the final BP value. In addition to performing routine blood testing that included lipid profiles, plasma Na, glucose levels, and blood/urine electrolytes, anthropometric measurements were obtained and a questionnaire was completed. The questionnaire included demographic information, personal history of cardiovascular diseases (angina pectoris, myocardial infarction, or stroke), and detailed personal and family histories of hypertension, overweight/obesity, diabetes mellitus, drug treatment, education, alcohol consumption, and cigarette smoking. The urinary Na excretion rate (UNa rate) was calculated as UNa*UV/24 h, where UNa* was the urinary concentration of Na, and UV was the urinary volume in 24 h. Body height and weight were used to calculate the body mass index (BMI; weight in kilograms divided by the height in meters squared). The diagnostic criteria for hypertension were defined as follows: a systolic blood pressure (SBP) ≥ 140 mmHg and/or a diastolic blood pressure (DBP) ≥ 90 mmHg or anti-hypertension treatment according to the WHO definition of hypertension in 1999. Non-hypertensive participants met the following criteria: no history of any anti-hypertensive medications and a SBP < 140 mmHg and a DBP < 90 mmHg.

Overweight and obesity were defined according to the criteria of WHO: normal weight, BMI < 25 kg/m^2^; overweight, 25 kg/m^2 ^≤ BMI ≤ 30 kg/m^2^; and obesity, BMI > 30 kg/m^2^.

Dyslipidemia was diagnosed according to the Guidelines for the Prevention and Control of Dyslipidemia in Chinese Adults as follows: total cholesterol (TC), ≥ 5.7 mmol/L; HDL-C, < 1.04 mmol/L; or low density lipoprotein-cholesterol (LDL-C), ≥ 3.37 mmol/L; or triglycerides (TG), ≥ 1.7 mmol/L.

Hyperglycemia was defined as a fasting blood glucose (FBG) ≥ 6.1 mmol/L or a 2-hour postprandial glucose (2 HPG) ≥ 7.8 mmol/L.

### Screening for genetic variations in *NEDD4L *in hypertensivepatients

We sequenced all exons and the promoter region of *NEDD4L*. Blood samples were obtained from 94 (47 males and 47 females) hypertensive patients, which were randomly chosen from the hypertensive group of the study population, and genomic DNA was isolated from peripheral blood leukocytes using a PAXgene Blood DNA kit (PreAnalytiX). All exons with their flanking sequences and approximately 500 bp of the upstream region of the promoter were directly sequenced by an ABI 3130Xl genetic analyzer (Applied Biosystems, Foster City, CA, USA) using 35 sets of primers, as described previously [18]. The obtained sequences were examined for the presence of variations using Sequencher 4.7 software (Gene Codes Corporation, Ann Arbor, MI, USA), followed by visual inspection. The A of the ATG of the initiator Met codon is denoted nucleotide +1. The nucleotide sequence [NCBI: NM-015277] was used as a reference sequence.

### Genotyping of representative SNPs in the general population

After considering the function and linkage disequilibrium relationships among the identified genetic variations, 3 common SNPs with a minor allele frequency of > 10% were selected as representatives for genotyping (Table 1). The TaqMan SNP Genotyping Assays were performed for genotyping using the method of Taq amplification in the 7900 HT Fast Real-Time PCR system (Applied Biosystems). The primers and probes (Applied Biosystems) used in the TaqMan SNP Genotyping Assays were chosen based on information available on the Applied Biosystems Inc. website http://myscience.appliedbiosystems.com. Finally, all of the three selected representative SNPs were successfully genotyped in 883 subjects participating in the study.

**Table 1:** Sequence variations in the promoter region and exons in Nedd4L identified in Kazakh hypertensive patients

SNP name	Region	Amino acid substitution	Allele 1 freq	Allele 2 freq	Flanking sequence	Typing	db SNP ID
-608C>G	promoter		0.9877	0.0123	cgccgcgccg[c/g]gctggaggcc		
-548G>A	promoter		0.9813	0.0187	cgcagcccgc[g/a]gcccgctcgc		
271420T>C	intron6		0.5989	0.4011	ttgtagtcag[t/c]gtattttcag	Taqman	rs2288774
271454A>G	intron6		0.7692	0.2308	caccacggtg[a/g]agaaggctga	Taqman	rs2288775
296921-296923delTTG	intron13		0.7381	0.2619	ttgcgttg[ttg/---]tttgggtt	Taqman	
297071G>A	exon14	R423R	0.9909	0.0091	AGATCCGCCG[G/A]CCTCGTAGCC		
312644T>G	intron18		0.9731	0.0269	tcttgtgtat[t/g]actgataacg		
312671C>T	intron18		0.9624	0.0376	caagggacaa[c/t]ggtgattgag		
325828A>G	intron22		0.9765	0.0235	atcaccgggc[a/g]ctcgtgtctt		
340782G>C	intron24		0.9892	0.0108	actccctgtt[g/c]cggagaaagc		
351555C>T	exon30	A944A	0.9944	0.0056	TTCTCATGGC[C/T]GTGGAAAATG		

Sequence variations were screened in 94 hypertensive patients. Taqman, The single nucleotide polymorphism (SNP) was successfully genotyped by the Taqman method. The A of the ATG of the initiator Met codon is denoted nucleotide +1, as recommended by the Nomenclature Working Group (Hum Mut 1998; 11:1--3). The nucleotide sequence [NCBI: NM-015277] wasused as a reference sequence.

### Statistical analysis

Data analyses were performed by SPSS for Windows (version 16; SPSS Inc., Chicago, IL, USA). Values are expressed as the means ± SD. The distribution of patient characteristics between the normotensive and hypertensive groups in the Kazakh general population was analyzed using a Student's *t*-test or a chi-square test. The differences in distributions of genotypes and alleles between the essential hypertension patients and control individuals were analyzed using a chi-square test. In addition, logistic regression analysis was performed to assess the contribution of the major risk factors (including smoking, drinking, age, and obesity). Covariate variance analysis was performed to compare the UNa rate and plasma Na level between the different genotypes after adjusting for age, smoking, and drinking. Two-way ANOVA (covariates: age and BMI) was used to assess interaction effects of gender and SNPs on BP traits, as well as interactions of smoking and SNPs. The frequency distribution of the haplotypes was calculated by chi-squared analysis. A case-control-based haplotype study, linkage disequilibrium, and the Hardy-Weinberg equilibrium were analyzed using SNPAlyze, version 7.0 Pro (DYNACOM Co. Ltd., Mobara, Japan). Statistical significance was established at *P *values < 0.05.

## Results

Eleven genetic variations in *NEDD4L *were identified by sequencing 94 hypertensive individuals, among which there were 3 common SNPs with a minor allele frequency > 10% (271420T>C, 271454A>G, and 296921-296923delTTG). 296921-296923delTTG is a new genetic common variation in *NEDD4L *which is not found in the NCBI SNP-database. No missense mutations in *NEDD4L *were identified, but we identified the following two synonymous mutations: R423 (297071G>A in exon14) with a minor allele frequency of 0.91%; and A944 (351555C>T in exon 30) with a minor allele frequency of 0.56% (Table 1). None of the variations were in tight linkage disequilibrium with an r^2 ^< 0.5 (Table 1).

Next, we looked for an association in the Kazakh general population between selected representative SNPs of *NEDD4L *and essential hypertension. Table 2 presents the clinical characteristics of the study participants. For males, females, and total participants, the following values were significantly higher for the essential hypertension patients than the control individuals: age, BMI, SBP, DBP, TC, TG, and FBG. There was no significant difference in HDL-C, UNa rate, and plasma Na level between the essential hypertension patients and the control individuals. The incidence of obesity, dyslipidemia, and hyperglycemia was significantly higher in the case group. There was no significant difference in the prevalence of smoking and drinking between the essential hypertension patients and control individuals.

**Table 2:** Basic characteristics of subjects in hypertensives and normotensives

	Total			Male			Female		
			
	Control (n = 500)	Case (n = 383)	*P*	Control (n = 204)	Case (n = 171)	*P*	Control (n = 296)	Case (n = 212)	*P*
Age (year)	42.04 ± 7.35	46.23 ± 7.63	< 0.001*	42.24 ± 7.48	46.11 ± 7.97	< 0.001*	41.90 ± 7.28	46.34 ± 7.37	< 0.001*
BMI (kg/m2)	25.27 ± 3.55	28.14 ± 4.50	< 0.001*	25.39 ± 3.23	28.52 ± 4.22	< 0.001*	25.18 ± 3.76	27.83 ± 4.70	< 0.001*
SBP (mmHg)	117.78 ± 11.11	150.60 ± 25.90	< 0.001*	119.43 ± 10.58	149.28 ± 25.67	< 0.001*	116.65 ± 11.34	151.67 ± 26.11	< 0.001*
DBP (mmHg)	78.28 ± 7.53	96.88 ± 10.92	< 0.001*	80.29 ± 6.24	97.03 ± 10.45	< 0.001*	76.88 ± 8.04	96.76 ± 11.32	< 0.001*
TC (mmol/L)	4.88 ± 1.00	5.14 ± 1.05	< 0.001*	5.09 ± 1.07	5.26 ± 1.03	0.111	4.84 ± 0.93	5.05 ± 1.06	0.001*
HDL (mmol/L)	1.47 ± 0.38	1.44 ± 0.39	0.244	1.31 ± 0.34	1.27 ± 0.31	0.244	1.58 ± 0.37	1.58 ± 0.40	0.927
TG (mmol/L)	1.00 ± 0.60	1.23 ± 0.74	< 0.001*	1.23 ± 0.75	1.53 ± 0.91	0.001*	0.85 ± 0.43	0.99 ± 0.46	0.001*
FBG (mmol/L)	4.83 ± 0.65	5.08 ± 1.35	0.001*	4.95 ± 0.63	5.21 ± 1.59	0.050	4.75 ± 0.65	4.98 ± 1.12	0.008*
UNa rate (uM/min)	163.78 ± 85.55	171.14 ± 87.57	0.260	182.64 ± 93.98	190.79 ± 88.33	0.439	150.31 ± 76.39	155.27 ± 83.90	0.536
Pl Na (mmol/L)	144.57 ± 8.98	144.74 ± 8.75	0.785	145.91 ± 9.32	145.09 ± 7.34	0.353	143.67 ± 8.66	144.45 ± 9.74	0.338
Life style									
Smoking (%)	22.8	24.3	0.606	55.4	52.6	0.593	0.3	1.4	0.176
Drinking (%)	21.6	21.1	0.871	52.0	44.4	0.147	0.7	2.4	0.109
Present illness									
Overweight (%)	49.9	74.3	0.000*	53.1	79.8	0.000*	47.7	69.8	0.000*
Dyslipidemia (%)	38.9	53.4	0.000*	60.0	68.8	0.078	24.7	41.0	0.000*
Hyperglycemia (%)	16.7	25.2	0.002*	15.5	23.5	0.057	17.4	26.6	0.014*

BMI; body mass index, SBP; systolic blood pressure, DBP; diastolic blood pressure, TC; total cholesterol, HDL-c; high-density lipoprotein cholesterol. TG; triglyceride, FBG; fasting blood glucose, Una rate; urinary Na excretion rate, PlNa; plasma Na. Values are mean ± SD or percentage. P < 0.05 was considered significant. The differences between case and control group were performed by t-test or *X*2 test.

* P < 0.05

Table 3 presents the distribution of the genotypes and alleles of the 3 common SNPs. The genotype distribution of each SNP was in Hardy-Weinberg equilibrium (data not shown). Power analyses revealed that our study had a power of 0.95 (for D-carrying genotype of 296921-296923delTTG) and 0.79 (for G-carrying genotype of rs2288775) to detect the difference across cases and controls in this study (α = 0.05). For total and male participants, the genotype distribution of the three SNPs did not differ significantly between the essential hypertension patients and the control individuals. For the females, the distribution of genotypes of rs2288775 differed significantly between the essential hypertension patients and the control individuals in the dominant model (AA *vs. *AG+GG, *P *= 0.040). The genotype distributions of 296921-296923delTTG were significantly different between the essential hypertension patients and the control individuals in the additive (*P *= 0.024) and recessive models (II+ID *vs. *DD, *P *= 0.007). The allelic frequencies of rs2288775 and 296921-296923delTTG were significantly different between the case and control groups (*P *= 0.038 and *P *= 0.028, respectively). Furthermore, there was a significant interaction between the *NEDD4L *genotype and gender (*P *for interaction: 0.045 for rs2288775 and 0.064 for 296921-296923delTTG), but no significant interaction between the *NEDD4L *genotype and smoking existed (*P *for interaction: 0.616 for rs2288775 and 0.447 for 296921-296923delTTG).

**Table 3:** Genotype and allele distributions in patients with essential hypertension and in control individuals

		Total			Male			Female		
				
SNPs	Genotype	Case (n = 383)	Control (n = 500)	*P*	Case (n = 171)	Control (n = 204)	*P*	Case (n = 212)	Control (n = 296)	*P*
rs2288774 n(%)										
Additive model	T/T	123 (32.5)	177 (35.8)	0.365	60 (35.7)	71 (35.1)	0.661	63 (30.0)	106 (36.2)	0.296
	C/T	173 (45.8)	228 (46.1)		76 (45.2)	99 (49.0)		97 (46.2)	129 (44.0)	
	C/C	82 (21.7)	90 (18.1)		32 (19.1)	32 (15.9)		50 (23.8)	58 (19.8)	
Dominant model	T/T	123 (32.5)	177 (35.8)	0.984	60 (35.7)	71 (35.1)	0.909	63 (30.0)	106 (36.2)	0.148
	T/C+C/C	255 (67.5)	318 (64.2)		108 (64.3)	131 (64.9)		147 (70.0)	187 (63.8)	
Recessive model	T/T+C/T	296 (78.3)	405 (81.9)	0.192	136 (80.9)	170 (84.1)	0.417	160 (76.2)	235 (80.2)	0.279
	C/C	82 (21.7)	90 (18.1)		32 (19.1)	32 (15.9)		50 (23.8)	58 (19.8)	
Allele	T	419 (55.4)	582 (58.8)	0.159	196 (58.3)	241 (59.7)	0.716	223 (53.1)	341 (58.2)	0.108
	C	337 (44.6)	409 (41.2)		140 (41.7)	163 (40.3)		197 (46.9)	245 (41.8)	
rs2288775 n(%)										
Additive model	A/A	210 (55.0)	302 (60.9)	0.189	100 (58.8)	122 (60.7)	0.901	110 (51.9)	180 (61.0)	0.114
	A/G	146 (38.2)	168 (33.9)		61 (35.9)	70 (34.8)		85 (40.1)	98 (33.2)	
	G/G	26 (6.8)	26 (5.2)		9 (5.3)	9 (4.5)		17 (8.0)	17 (5.8)	
Dominant model	A/A	210 (55.0)	302 (60.9)	0.078	100 (58.8)	122 (60.7)	0.714	110 (51.9)	180 (61.0)	0.040*
	A/G+G/G	172 (45.0)	194 (39.1)		70 (41.2)	79 (39.3)		102 (48.1)	115 (39.0)	
Recessive model	A/A+AG	356 (93.2)	470 (94.8)	0.948	161 (94.7)	192 (95.5)	0.715	195 (92.0)	278 (94.2)	0.316
	G/G	26 (6.8)	26 (5.2)		9 (5.3)	9 (4.5)		17 (8.0)	17 (5.8)	
Allele	A	566 (74.1)	772 (77.8)	0.065	261 (76.8)	314 (78.1)	0.662	305 (71.9)	458 (77.6)	0.038*
	G	198 (25.9)	220 (22.2)		79 (23.2)	88 (21.9)		119 (28.1)	132 (22.4)	
296921-296923 delTTG n(%)										
Additive model	I/I	186 (48.8)	258 (52.3)	0.217	91 (53.5)	111 (54.7)	0.674	95 (45.0)	147 (50.7)	0.024*
	I/D	155 (40.7)	199 (40.4)		69 (40.6)	76 (37.4)		86 (40.8)	123 (42.4)	
	D/D	40 (10.5)	36 (7.3)		10 (5.9)	16 (7.9)		30 (14.2)	20 (6.9)	
Dominant model	I/I	186 (48.8)	258 (52.3)	0.303	91 (53.5)	111 (54.7)	0.824	95 (45.0)	147 (50.7)	0.210
	D/D+I/D	195 (51.2)	235 (47.7)		79 (46.5)	92 (45.3)		116 (55.0)	143 (49.3)	
Recessive model	I/I+I/D	341 (89.5)	457 (92.7)	0.096	160 (94.1)	187 (92.1)	0.450	181 (85.8)	270 (93.1)	0.007*
	D/D	40 (10.5)	36 (7.3)		10 (5.9)	16 (7.9)		30 (14.2)	20 (6.9)	
Allele	I	527 (69.2)	715 (72.5)	0.125	251 (73.8)	298 (73.4)	0.896	276 (65.4)	417 (71.9)	0.028*
	D	235 (30.8)	271 (27.5)		89 (26.2)	108 (26.6)		146 (34.6)	163 (28.1)	

* *P *< 0.05

After adjusting for confounding factors (age, smoking, drinking, and obesity), multivariate logistic regression analysis also showed that rs2288775 (in the dominant model) and 296921-296923delTTG (in the recessive model) were significantly associated with hypertension (rs2288775: OR = 1.479, 95% CI = 1.011-2.064, *p *= 0.044; 296921-296923delTTG: OR = 1.908, 95% CI = 1.020-3.568, *p *= 0.043; Table 4).

**Table 4:** Odds ratios and 95% confidence intervals for two variations of NEDD4L gene associated with essential hypertension in female after adjusting smoking, drinking, age and obesity

polymorphism	genotype	odds ratios	95% confidence interval	*P*
rs2288775	A/A	1		
	A/G+G/G	1.479	1.011-2.064	0.044*
296921-296923delTTG	II+ID	1		
	D/D	1.908	1.020-3.568	0.043*

* *P *< 0.05

In the haplotype-based case-control analysis, haplotypes were established in three representative common SNPs (Table 5). For the total population and males, the distribution of the haplotypes was not significantly different between the essential hypertension patients and the control individuals. For females, the frequency of D-C-G haplotype (established by 296921-296923delTTG, rs2288774, and rs2288775) were significantly higher for the essential hypertension patients than for the control individuals (*P *= 0.020).

**Table 5:** Haplotype analysis in patients with essential hypertension and control participants

Haplotype				Total			Male			Female		
			
	296921-3delTTG	rs2288774	rs2288775	Case (n = 383)	Control (n = 500)	*P*	Case (n = 171)	Control (n = 204)	*P*	Case (n = 212)	Control (n = 296)	*P*
H1	I	T	A	0.526	0.558	0.190	0.562	0.565	1.000	0.497	0.554	0.087
H2	D	C	G	0.229	0.192	0.058	0.190	0.185	0.913	0.260	0.197	0.020*
H3	I	C	A	0.136	0.140	0.856	0.140	0.135	0.924	0.134	0.143	0.718
H4	D	C	A	0.050	0.053	0.825	0.044	0.055	0.595	0.054	0.052	0.899

* *P *< 0.05

Table 6 presents the comparison of the UNa rate and plasma Na between the different genotypes by covariate variance analysis, adjusting for age, smoking, and drinking. For females and the total participants, the UNa rate was significantly lower for the DD than the I/I+I/D individuals (*P *= 0.032 and *P *= 0.027, respectively). There was no significant difference in the plasma Na level between the genotypes.

**Table 6:** Comparing of urinary Na excretion rate and plasma Na between different genotypes by Covariate variance analysis adjusting for age, smoking, and drinking.

	296921-296923delTTG			Rs2288775		
		
	I/I+I/D	D/D	*P*	A/A	A/G+G/G	*P*
UNa rate (uM/min)						
Total	169.27 ± 87.71	143.92 ± 69.00	0.027*	167.69 ± 86.10	166.86 ± 87.43	0.954
Male	186.75 ± 92.03	180.50 ± 83.87	0.798	182.82 ± 91.23	193.80 ± 91.83	0.278
Female	155.50 ± 81.69	126.49 ± 53.47	0.032*	156.21 ± 80.32	147.25 ± 78.73	0.233
Pl Na (mmol/L)						
Total	144.69 ± 8.83	144.11 ± 9.62	0.496	144.67 ± 8.46	144.58 ± 9.49	0.900
Male	145.67 ± 8.61	143.65 ± 6.02	0.238	145.22 ± 7.08	145.98 ± 10.27	0.330
Female	143.94 ± 8.93	144.34 ± 11.09	0.846	144.25 ± 9.36	143.63 ± 8.83	0.471

Una rate; urinary Na excretion rate, PlNa; plasma Na. * P < 0.05

The associations between the SNPs and overweight, dyslipidemia, hyperglycemia, and central obesity were also examined, and no significant associations were found (Additional file 1).

## Discussion

In this study by systemically screening variations of *NEDD4L *we did not identify any functional mutations in *NEDD4L*. By studying the associations of 3 representative common SNPs with hypertension, we first report that two common SNPs (296921-296923delTTG and rs2288775) were significantly associated with essential hypertension in Kazakh females.

It has been reported that NEDD4L plays an important role in regulating BP [5-8]. Importantly, by studying some functional SNPs of *NEDD4L *chosen from the public genetic database, several studies have recently demonstrated that genetic variations of *NEDD4L *are associated with hypertension [9,10,19-21]. So *NEDD4L *is an important candidate gene for hypertension, and there is a need to study its relationship to hypertension comprehensively from a genetic point of view. In this study, we systemically studied the association of genetic variations of *NEDD4L *with essential hypertension in Xinjiang Kazakh using the research strategy that we first screened genetic variations of *NEDD4L *and then studies the relationship of the representative SNPs to hypertension. The research strategy was selected based on the following: a) the HapMap project does not provide genetic information for Xinjiang Kazakh, so we could not use the Tag-SNP specific for Kazakh in this study; and b) by sequencing the functional regions with their flanking sequences of *NEDD4L *in 94 Kazakh hypertensives, we found not only common and rare SNPs or mutations, both of which are considered to contribute to the pathogenesis of essential hypertension, but also novel or race-specific genetic variations.

In this study, we did not find any missense mutations in *NEDD4L*. This may be indicative of the relatively high conservation of NEDD4L and the importance of this molecule in regulating salt and water balance and BP. A novel common SNP (296921-296923delTTG) in *NEDD4L*, which is not found in the NCBI SNP-database, was significantly associated with essential hypertension in Kazakh females. Another polymorphism, rs2288745, located at the 100 bp downstream exon 6, was also positive in Kazakh females with essential hypertension. Since significant associations of the two common SNPs with essential hypertension were obtained in the multivariable analysis with adjustment for confounding risk factors, including age, obesity, and lifestyle (current smoking and drinking), the G allele of rs2288745 may be an independent risk factor for hypertension and the I allele of 296921-296923delTTG may be an independent protective factor for hypertension. In the present study, we genotyped three SNPs that are not in tight linkage disequilibrium, therefore we had to perform a correction for multiple testing. After correction by the Bonferroni method (corrected *P *= 0.017), no SNPs were significantly associated with hypertension after adjustment for confounding factors. Haplotype analysis is considered to be a good method used in genetic studies for complex diseases while avoiding problems induced by multiple testing. In this study, one haplotype of *NEDD4L *was significantly associated with essential hypertension. Moreover, power analyses revealed that our study had a power of 0.95 (for the D-carrying genotype of 296921-296923delTTG) and 0.79 (for the G-carrying genotype of rs2288775) to detect the difference across cases and controls in this study (α = 0.05). Furthermore, the distribution of all polymorphisms is under Hardy-Weinberg equilibrium (data not shown), which suggests the results of this study are unlikely to be biased by population stratification or admixture for essential hypertension. Together, it is strongly suggested that rs2288745 and 296921-296923delTTG may be associated with essential hypertension.

The mechanisms by which the common SNPs (296921-296923delTTG and rs2288775) might contribute to hypertension are unknown. The two common SNPs are located in intronic regions. It is generally assumed that the sequence of any given intron is junk DNA with no biological function. More recently, however, this is being disputed. Introns contain several short sequences that are important for efficient splicing, such as acceptor and donor sites at either end of the intron, as well as a branch point site, which are required for proper splicing by the spliceosome. Further studies are critically needed to determine whether the polymorphisms of 296921-296923delTTG and rs2288775 affect the splicing of mRNA of NEDD4L. In addition, these two SNPs may be mere genetic markers and it may be in linkage disequilibrium with another functional variation within the *NEDD4L *and other functional polymorphisms may play more important roles in hypertension. Rs4149601 (16229 bps from exon 2) of *NEDD4L *is associated with hypertension in African Americans and Caucasians and rs3865418 (4106 bps from exon 13) has been shown to have a "flip-flop" association with hypertension in two Caucasian samples [10]. The region harboring these two SNPs was not included by our sequencing method, thus a greater density of genotyping around *NEDD4L *or the two common SNP sites is needed.

In the HapMap database, we found genotype data for rs2288774, but not for rs2288775. The frequencies of minor alleles of rs2288774 are different between various ethnicities (Japanese, Chinese Han, Caucasians, blacks, and Xinjiang Kazakh [from the data of the present study]; 31.0%, 32.0%, 48.0%, 33.0%, and 41.2%, respectively). This strongly indicates that the distributions of genotypes of common SNPs might be different between ethnicities. As association studies are not consistently reproducible as a result of false positives, false negatives, and problems with true variability in association between different populations [22], the associations of 296921-296923delTTG and rs2288775 with essential hypertension must be re-examined in another population.

The present population-based case-control study also showed gender-specific (for females only) significant differences in genetic markers between essential hypertension patients and control individuals. Moreover, there was a significant gene-by-gender interaction on BP traits in this study. Several studies have reported gender-specific effects of gene variants and gene-by-gender interactions in human hypertension [23-26]. These data support our findings. Regarding the mechanisms by which two common SNPs (296921-296923delTTG and rs2288775) might contribute to essential hypertension in only females, it has been reported that NEDD4L expression is down-regulated in prostate cancer and up-regulated following androgen treatment [27,28], suggesting that the variations of *NEDD4L *are associated with androgen-mediated gender differences in essential hypertension. Unfortunately, we could not obtain data on plasma sex hormones as we were not able to obtain informed consent to collect blood samples for the purpose of such measurements. It appears that two common SNPs (296921-296923delTTG and rs2288775) in the *NEDD4L *and/or neighboring genes are associated with essential hypertension in females. Furthermore, there is a significant difference between males and females for self-reported smoking status in this study population (> 50% of males and only about 1% of females report being smokers). Is it apparent that the gene-by-gender interaction is only a proxy for an underlying gene-by-smoking interaction? Therefore, we next assessed the gene-by-smoking interaction effects on BP traits, but no significant interaction was found in this study. Together, it is suggested that the gender-specific findings in this study are reliable.

## Conclusion

In summary, two common SNPs of *NEDD4L *(296921-296923delTTG and rs2288775), were found to be associated with essential hypertension in Kazakh females. This reconfirms that genetic variations of *NEDD4L *might be involved in the pathogenesis of essential hypertension.

## Competing interests

The authors declare that they have no competing interests.

## Authors' contributions

NFL, HMW and JY carried out the molecular genetic studies and drafted the manuscript. YYG, WLL, and JHC participated in the sequence alignment. LZ and JH participated in the design of the study and performed the statistical analysis. NFL conceived of the study, participated in its design and coordination. All authors read and approved the final manuscript.

## Pre-publication history

The pre-publication history for this paper can be accessed here:

http://www.biomedcentral.com/1471-2350/10/130/prepub

## Supplementary Material

Additional file 1 **Genotype distributions in obesity, hyperglycemia, dyslipidemia patients and in control individuals**. The associations between the SNPs and overweight, dyslipidemia, hyperglycemia, central obesity were also examined respectively, and no significant associations were found in this study. Click here for file
