# Supplementary material for: Genetic variation of NEDD4L is associated with essential hypertension in female Kazakh general population: a case-control study
Source: BMC Med Genet. 2009 Dec 9;10:130. doi: 10.1186/1471-2350-10-130 (PMC2801499; doi:10.1186/1471-2350-10-130)
Supplement: Additional file 1 — Genotype distributions in obesity, hyperglycemia, dyslipidemia patients and in control individuals. The associations between the SNPs and overweight, dyslipidemia, hyperglycemia, central obesity were also examined respectively, and no significant associations were found in this study. [file 1471-2350-10-130-S1.DOC]

| ***Additional file 1*** *Genotype distributions in obesity, hyperglycemia, dyslipidemia patients and in control individuals* | | | | | | | | | | | | | | | | | | |
| --- | --- | --- | --- | --- | --- | --- | --- | --- | --- | --- | --- | --- | --- | --- | --- | --- | --- | --- |
|  |  | Overweight and obesity | | | | | | | |  | Hyperglycemia | | | | | | | |
|  |  | Total | |  | Male | |  | Female | |  | Total | |  | Male | |  | Female | |
|  |  | Case | Control |  | Case | Control |  | Case | Control |  | Case | Control |  | Case | Control |  | Case | Control |
| 296921-296923delTTG | |  |  |  |  |  |  |  |  |  |  |  |  |  |  |  |  |  |
|  | I/I | 295 | 199 |  | 141 | 73 |  | 154 | 126 |  | 348 | 87 |  | 152 | 43 |  | 196 | 44 |
|  | I/D | 185 | 123 |  | 80 | 48 |  | 105 | 75 |  | 269 | 71 |  | 113 | 24 |  | 156 | 47 |
|  | D/D | 37 | 15 |  | 13 | 18 |  | 24 | 10 |  | 61 | 15 |  | 24 | 2 |  | 37 | 13 |
|  | *P* | 0.270 | |  | 0.658 | |  | 0.208 | |  | 0.947 | |  | 0.176 | |  | 0.311 | |
| rs2288774 | |  |  |  |  |  |  |  |  |  |  |  |  |  |  |  |  |  |
|  | T/T | 255 | 172 |  | 130 | 64 |  | 125 | 108 |  | 231 | 59 |  | 95 | 28 |  | 136 | 31 |
|  | C/T | 209 | 139 |  | 89 | 53 |  | 120 | 86 |  | 306 | 83 |  | 137 | 32 |  | 169 | 51 |
|  | C/C | 51 | 24 |  | 17 | 9 |  | 34 | 15 |  | 143 | 28 |  | 57 | 6 |  | 86 | 22 |
|  | *P* | 0.386 | |  | 0.712 | |  | 0.118 | |  | 0.394 | |  | 0.088 | |  | 0.534 | |
| rs2288775 |  |  |  |  |  |  |  |  |  |  |  |  |  |  |  |  |  |  |
|  | A/A | 170 | 117 |  | 83 | 41 |  | 87 | 76 |  | 400 | 98 |  | 169 | 45 |  | 231 | 53 |
|  | A/G | 237 | 156 |  | 108 | 65 |  | 129 | 91 |  | 240 | 66 |  | 104 | 21 |  | 136 | 45 |
|  | G/G | 106 | 63 |  | 42 | 20 |  | 64 | 43 |  | 43 | 9 |  | 17 | 1 |  | 26 | 8 |
|  | *P* | 0.761 | |  | 0.635 | |  | 0.483 | |  | 0.702 | |  | 0.213 | |  | 0.265 | |

| ***Additional file 1*** *continue* | | | | | | | | | | | | | | | | |
| --- | --- | --- | --- | --- | --- | --- | --- | --- | --- | --- | --- | --- | --- | --- | --- | --- |
| Hyperglycemia | | | | | | | |  | central obesity | | | | | | | |
| Total | |  | Male | |  | Female | |  | Total | |  | Male | |  | Female | |
| Case | Control |  | Case | Control |  | Case | Control |  | Case | Control |  | Case | Control |  | Case | Control |
|  |  |  |  |  |  |  |  |  |  |  |  |  |  |  |  |  |
| 205 | 237 |  | 134 | 66 |  | 71 | 171 |  | 212 | 214 |  | 91 | 103 |  | 121 | 111 |
| 160 | 192 |  | 90 | 53 |  | 70 | 139 |  | 176 | 170 |  | 64 | 78 |  | 112 | 92 |
| 31 | 45 |  | 13 | 13 |  | 18 | 32 |  | 46 | 29 |  | 14 | 12 |  | 32 | 17 |
| 0.664 | |  | 0.216 | |  | 0.507 | |  | 0.178 | |  | 0.709 | |  | 0.243 | |
|  |  |  |  |  |  |  |  |  |  |  |  |  |  |  |  |  |
| 141 | 157 |  | 90 | 39 |  | 51 | 118 |  | 145 | 141 |  | 58 | 66 |  | 87 | 75 |
| 183 | 217 |  | 110 | 64 |  | 73 | 153 |  | 194 | 198 |  | 74 | 99 |  | 120 | 99 |
| 71 | 100 |  | 35 | 28 |  | 36 | 72 |  | 95 | 73 |  | 35 | 27 |  | 60 | 46 |
| 0.473 | |  | 0.145 | |  | 0.840 | |  | 0.300 | |  | 0.179 | |  | 0.897 | |
|  |  |  |  |  |  |  |  |  |  |  |  |  |  |  |  |  |
| 234 | 276 |  | 145 | 75 |  | 89 | 201 |  | 248 | 245 |  | 101 | 113 |  | 147 | 132 |
| 140 | 172 |  | 81 | 48 |  | 59 | 124 |  | 155 | 152 |  | 56 | 72 |  | 99 | 80 |
| 21 | 31 |  | 9 | 9 |  | 12 | 22 |  | 34 | 17 |  | 11 | 7 |  | 23 | 10 |
| 0.742 | |  | 0.375 | |  | 0.835 | |  | 0.078 | |  | 0.373 | |  | 0.176 | |
